# Frailty Assessment to Improve Risk Stratification in Elderly Patients Undergoing Elective Cardiac Surgery

**DOI:** 10.21470/1678-9741-2023-0182

**Published:** 2025-03-13

**Authors:** Kamile Ozeren, Ahmet Can Topcu, Ilyas Kayacioglu

**Affiliations:** 1 Department of Cardiovascular Surgery, Dr. Siyami Ersek Thoracic and Cardiovascular Surgery Education and Research Hospital, Istanbul, Turkey; 2 Department of Cardiovascular Surgery, Kosuyolu High Specialization Education and Research Hospital, Istanbul, Turkey

**Keywords:** Aged, Prognosis, Frailty, Serum Albumin, Patient Discharge, Cardiac Surgical Procedures, Risk Factors, Morbidity

## Abstract

**Introduction:**

Frailty is a biological syndrome of the elderly characterized by decreased physiological reserve and weakened response to stressors. Most cardiac surgical risk models incorporate chronologic age as a risk parameter, but not frailty. We aimed to identify the frailty assessment tool with the highest prognostic value to predict postoperative adverse outcomes in elderly patients undergoing cardiac surgery and to investigate whether addition of a frailty parameter to cardiac surgical risk models would increase predictive power.

**Methods:**

This is a single-center, prospective, observational study. Consecutive adults, undergoing elective cardiac surgery between January and May 2020, were included. The European System for Cardiac Operative Risk Evaluation II (EuroSCORE II) and Society of Thoracic Surgeons risk scores were calculated. Fried Scale, Short Physical Performance Battery, Clinical Frailty Scale, and serum albumin were used for frailty assessment. Patients were followed-up for 30 days postoperatively or until discharge. Primary endpoint was a composite of mortality and major morbidity.

**Results:**

One hundred sixty-four patients were included (34.76% women, median age 70 years [interquartile range, 67-74]. EuroSCORE II and albumin were the only tools significantly associated with the primary endpoint (*P*=0.045 and *P*=0.031, respectively). Model created by combination of EuroSCORE II and albumin was not associated with the primary outcome (*P*=0.571), however EuroSCORE II’s R-squared value increased from 0.07 to 0.144 after addition of albumin.

**Conclusion:**

Addition of albumin measurement as a frailty marker to EuroSCORE II has the potential to improve EuroSCORE II’s ability to predict early postoperative mortality/morbidity in elderly patients undergoing cardiac surgery.

## INTRODUCTION

World population is aging, and so is the cardiac surgery population. Patients ≥ 65 years of age make up more than half of the adult cardiac surgery population^[1]^. Advanced age is a significant risk factor for postoperative adverse outcomes, however, chronological age does not always reflect biological age^[2]^. The most common cardiac surgical risk models incorporate chronologic age as a risk parameter, but not patients’ frailty status or biological age^[3,4]^. Frailty is a biological syndrome characterized by decreased physiological reserve and weakened response to stressors, associated with advanced age^[5,6]^. There is no gold standard definition of frailty, and its prevalence varies considerably depending on which assessment tool is used^[6,7]^. The phenomenon of frailty is widely studied in patients over the age of 65 and should be included in the risk assessment process of surgical candidates of this age group^[6,7]^.

We aimed to identify the frailty assessment tool with the highest prognostic value to predict postoperative adverse outcomes in elderly patients undergoing cardiac surgery and to investigate whether addition of a frailty parameter to cardiac surgical risk models would increase the predictive power of these models.

## METHODS

### Study Design and Participants

This is a single-center, prospective, observational study. The study protocol was approved by Haydarpasa Numune Education and Research Hospital Clinical Research Ethics Committee on July 22, 2019 (ID: HNEAH-KAEK 2019/KK/76). A written informed consent was obtained from each participant. All procedures related to the study were in accordance with the ethical standards of the 1964 Helsinki Declaration. The study protocol was registered on ClinicalTrials.gov on December 10, 2019 (NCT04191915). Consecutive adult patients, scheduled to undergo elective cardiac surgery between January 15 and May 15, 2020, at Dr. Siyami Ersek Thoracic and Cardiovascular Surgery Education and Research Hospital, were assessed for enrollment. Inclusion criteria were (1) patient’s age ≥ 65 years and (2) patient undergoing coronary artery bypass grafting (CABG), heart valve surgery, or combined CABG and valve surgery. Patients were excluded if they (1) were undergoing emergency surgery; (2) refused to participate; (3) had hemodynamic instability; or (4) had neuropsychiatric disorders.

### Cardiac Surgical Risk Assessment

The European System for Cardiac Operative Risk Evaluation II (EuroSCORE II) and the Society of Thoracic Surgeons (STS) Adult Cardiac Surgery Risk Model were used to assess operative risk^[3,4]^. STS scores were calculated to estimate both predicted risk of mortality (STS-PROM) and predicted risk of mortality and major morbidity (STS-PROMM)^[4]^.

### Frailty Assessment

Frailty tests were performed by a single operator (K.O.) one day prior to surgery. Several widely accepted and commonly used tests, Fried scale, Short Physical Performance Battery (SPPB), Clinical Frailty Scale (CFS), serum albumin, and Katz index were utilized for frailty assessment. Fried scale’s and SPPB’s components were also individually tested for their ability to predict outcome. Five components of Fried’s frailty phenotype were examined as described by Fried et al.:^[5]^ shrinking (unintentional weight loss), weakness (low hand grip strength), self-reported exhaustion, slowness (low gait speed), and low physical activity (Supplementary Table 1). Patients were diagnosed as frail if they met ≥ 3 criteria. The SPPB, another frailty assessment tool based on phenotypic approach, is comprised of three components: slowness, weakness, and balance^[8]^. Standing balance, four-meter walk, and chair rise tests were used to measure balance, gait speed, and weakness, respectively, as described by Guralnik et al.^[8]^ (Supplementary Table 2). Each component was scored 0 to 4, and patients received a total score of 0 to 12. An SPPB score of 0 to 8 classified patients as frail^[9]^. Current version of the CFS^[10]^, which was originally designed by Rockwood et al. to represent and correlate with a frailty index, was administered to score patients’ frailty degree on a scale of 1 (very fit) to 9 (terminally ill) based on their functional status. The most common cutoff value of ≥ 5 was used to classify patients as frail^[11]^. Serum albumin was recorded as a biomarker of frailty^[12,13]^.

**Supplementary Table 1:** Fried scale^[5]^.

Phenotypic criteria	Measurement	Point
1. Shrinking	Unintentional weight loss ≥ 4.5 kg or ≥ 5% of body weight in the past 12 months	1 point
2. Weakness	Average of hand grip strength measured 3 times using a digital hand dynamometer:	1 point
	Men	
	BMI ≤ 24 and hand grip strength ≤ 29 kg	
	BMI = 24.1-26 and hand grip strength ≤ 30 kg	
	BMI = 26.1-28 and hand grip strength ≤ 30 kg	
	BMI > 28 and hand grip strength ≤ 32 kg	
	Women	
	BMI ≤ 23 and hand grip strength ≤ 17 kg	
	BMI = 23.1-26 and hand grip strength ≤ 17.3 kg	
	BMI = 26.1-29 and hand grip strength ≤ 18 kg	
	BMI > 29 and hand grip strength ≤ 21 kg	
3. Slowness	5-meter gait speed measured at a regular pace:	1 point
	Men	
	Height ≤ 173 cm and gait speed ≤ 0.65 m/sec	
	Height > 173 cm and gait speed ≤ 0.76 m/sec	
	Women	
	Height ≤ 159 cm and gait speed ≤ 0.65 m/sn	
	Height > 159 cm and gait speed ≤ 0.76 m/sn	
4. Low physical activity	Questioning of weekly energy expenditure using a short version of Minnesota Leisure Time Activity	1 point
	Questionnaire:	
	Men < 383 kcal/week	
	Women < 270 kcal/week	
5. Exhaustion	An answer of “often (≥ 3 days)” to any of the following questions:	1 point
	“In the last week, how often did you feel that everything you did was an effort?”	
	“In the last week, how often did you feel that you could not get going?”	

≥ 3 points: frail; BMI=body mass index

**Supplementary Table 2:** Short Physical Performance Battery^[8]^.

**1. Balance test (0 to 4 points)**			
**Feet together**	**Semi-tandem**	**Tandem**	
< 10 sec.	-	-	0 points
10 sec.	< 10 sec.	-	1 point
10 sec.	10 sec.	< 3 sec.	2 points
10 sec.	10 sec.	3 to 9.99 sec.	3 points
10 sec.	10 sec.	10 sec.	4 points
**2. 4-meter gait speed test (0 to 4 points)**			
Unable to perform			0 points
> 8.7 sec.			1 point
6.21 to 8.7 sec.			2 points
4.82 to 6.2 sec.			3 points
< 4.82 sec.			4 points
**3. Chair rise test**			
Unable to perform			0 points
≥ 16.7 sec.			1 point
13.7 to 16.6 sec.			2 points
11.2 to 13.6 sec.			3 points
≤ 11.1 sec.			4 points

0 to 8: frail

Katz Index of Independence in Activities of Daily Living was applied to participants. One point was given for self-reported performance of each of the following activities without supervision, direction, or personal assistance: bathing, dressing, toileting, transferring, continence, feeding. Each participant obtained a score of 0 (very dependent) to 6 (independent).

### Follow-up and Study Endpoints

Patients were followed-up for 30 days after surgery or during index hospitalization (whichever is longer).

The primary endpoint was a composite of operative mortality and/or any major morbidity including stroke, acute renal failure, prolonged ventilation, deep sternal wound infection, and re-operation, as described by the STS^[4]^. Participants who experienced any of the abovementioned adverse outcomes were considered to have reached the primary endpoint. Operative mortality included all deaths, regardless of cause. Stroke was defined as global or focal neurological dysfunction caused acutely by ischemia or hemorrhage and lasting for at least 24 hours. Acute renal failure was defined as occurrence of any of the following: new-onset need for renal replacement therapy, a serum creatinine level of ≥ 4 mg/dl with an increase of ≥ 0.5 mg/dl from baseline, a ≥ 3-fold increase in creatinine from baseline, a urine output of ≤ 0.3 ml/kg/hour for ≥ 24 hours, or anuria for ≥ 12 hours. Prolonged ventilation was defined as reintubation or mechanical ventilation ≥ 24 hours.

Secondary endpoints were prolonged hospital stay (> 14 days), prolonged intensive care unit stay (> 48 hours), and hospital readmission for any reason.

### Statistical Analysis

Power analysis was performed to estimate sample size using G*Power software v3.1.7. The primary endpoint of composite mortality and/or major morbidity was tested with a predicted incidence of 19.3% as previously reported by Afilalo et al.^[2]^ in a similar patient population. At least 150 participants were needed to achieve an 80% (Beta=0.2) power at the 5% (Alpha=0.05) level of significance according to Cohen’s kappa coefficient.

IBM SPSS Statistics for Windows (version 28.0, Armonk, NY: IBM Corp.) was used for statistical analyses. Categorical variables were presented as numbers (n) and proportions (%). Continuous variables were presented as median (interquartile range [IQR]). Normality of quantitative data was assessed with Shapiro-Wilk test, Q-Q plots, and histograms. Independent samples *t*-test was used to compare normally distributed variables. Mann-Whitney U test was used to compare non-normally distributed variables. Qualitative data were analyzed with Pearson’s chi-squared test. Fleiss’ kappa and Cohen’s kappa were used to assess agreement of frailty tests on identifying patients as frail. Missing data was handled using pairwise deletion. Frailty assessment results were analyzed as categorical variables except for albumin. Albumin measurement was treated as a continuous variable, and the optimal cutoff with the highest sensitivity and specificity for the primary endpoint was identified using univariate logistic regression analysis. Multinominal logistic regression analysis was performed to create predictive models. Receiver operating characteristic curves were plotted from logistic regression models, and areas under curves were compared using DeLong’s method. Risk ratio with 95% confidence interval was calculated to assess relative risk of being frail to experience the primary outcome. A two-sided *P*-value of < 0.05 was considered statistically significant.

## RESULTS

A total of 164 patients were included (57 women, 34.76%). Median patient age was 70 years (range, 65-85; IQR, 67-74). One hundred six (64.63%) patients underwent isolated CABG, 38 (23.17%) underwent isolated valve surgery, and 20 (12.2%) underwent combined CABG and valve surgery. The study population had a median EuroSCORE II of 2.27% (IQR, 1.47-3.98), STS-PROM of 1.4% (IQR, 0.92-2.41), and STS-PROMM of 10.28% (IQR, 7.76-15.04) (Table 1).

**Table 1:** Demographics, comorbidities, and operative details.

Age (years), median (IQR)	70 (67; 74)
Female sex, n (%)	57 (34.76)
Body mass index (kg/m^2^), median (IQR)	28.4 (25.7; 32.5)
Left ventricular ejection fraction (%), median (IQR)	55 (45; 60)
Glomerular filtration rate (ml/dk/1,73 m^2^), median (IQR)	71.7 (58.1; 86)
Creatinine (mg/dl), median (IQR)	0.995 (0.83; 1.17)
NYHA class, n (%)	
II	109 (66.46)
III	55 (33.54)
Current smoker, n (%)	21 (12.8)
Hypertension, n (%)	89 (54.27)
Diabetes, n (%)	65 (39.63)
Chronic renal disease, n (%)	47 (28.66)
Chronic obstructive pulmonary disease, n (%)	73 (44.51)
Arrhythmia, n (%)	22 (13.41)
Carotid artery disease, n (%)	25 (15.34)
Peripheral artery disease, n (%)	6 (3.66)
History of stroke, n (%)	18 (10.98)
Previous myocardial infarction, n (%)	56 (34.15)
Previous cardiac surgery, n (%)	8 (4.88)
EuroSCORE II (%), median (IQR)	2.27 (1.47; 3.98)
STS-PROM (%), median (IQR)	1.4 (0.92; 2.41)
STS-PROMM (%), median (IQR)	10.28 (7.76; 15.04)
Operation, n (%)	
CABG	106 (64.63)
Valve surgery	38 (23.17)
Combined procedure	20 (12.2)

CABG=coronary artery bypass grafting; EuroSCORE II=European System for Cardiac Operative Risk Evaluation II; IQR=interquartile range; NYHA=New York Heart Association; STS-PROM=Society of Thoracic Surgeons-predicted risk of mortality; STS-PROMM=Society of Thoracic Surgeons-predicted risk of mortality and morbidity

All participants completed follow-up (100%). Primary endpoint, the composite of postoperative mortality and/or any major morbidity, was observed in 41 (25%) patients. Operative mortality rate was 9.1% with 15 deaths. Prolonged hospital and intensive care unit stay occurred in 26 (15.9%) and 35 (25.3%) patients, respectively. Eighteen (11%) hospital survivors were rehospitalized within the follow-up period (Table 2).

**Table 2:** Study endpoints.

Primary composite endpoint, n (%)	41 (25)
Mortality, n (%)	15 (9.1)
Stroke, n (%)	6 (3.7)
Acute renal failure, n (%)	9 (5.5)
Prolonged mechanical ventilation, n (%)	18 (11)
Deep sternal wound infection, n (%)	11 (6.7)
Reoperation, n (%)	18 (11)
Prolonged hospital stay, n (%)	26 (15.9)
Prolonged intensive care unit stay, n (%)	35 (21.3)
Hospital readmission for any reason, n (%)	18 (11)

Demographics were not significantly associated with postoperative adverse outcomes (Table 3). Patients who experienced mortality and/or major morbidity were slightly older than those who did not; median age 71 (IQR, 69-75) *vs.* 69 (IQR, 67-73) years (*P*=0.048), respectively (Table 3). However, a point-biserial analysis did not demonstrate a significant correlation between age and primary outcome (r=0.151, *P*=0.053).

**Table 3:** Relationship of age, body mass index, and sex with endpoints.

	n (%)	Age (years), median (IQR)	BMI (kg/m^2^), median (IQR)	Sex	
				Female, n (%)	Male, n (%)
**Primary endpoint**					
No	123 (75)	69 (67; 73)	28.71 (25.97; 32.76)	40 (70.2)	83 (77.6)
Yes	41 (25)	71 (69; 75)	27.55 (25.06; 30.86)	17 (29.8)	24 (22.4)
*P*-value		*P*=0.048^a^	*P*=0.321^a^	*P*=0.298*^b^*	
**Prolonged hospital stay**					
No	138 (84)	69 (67; 74)	28.37 (25.4; 32.45)	46 (80.7)	92 (86)
Yes	26 (16)	71.5 (68; 74)	29 (26.12; 32.85)	11 (19.3)	15 (14)
*P*-value		*P*=0.114^a^	*P*=0.254^a^	*P*=0.378^b^	
**Prolonged intensive care unit stay**					
No	129 (79)	69 (67; 73)	28.64 (25.59; 32.88)	46 (80.7)	83 (77.6)
Yes	35 (21)	71 (68.5; 75)	27.78 (25.99; 31.44)	11 (19.3)	24 (22.4)
P-value		*P*=0.052^a^	*P*=0.709^a^	*P*=0.641^b^	
**Hospital readmission**					
No	146 (89)	70 (67; 74)	28.37 (25.88; 32.02)	53 (93)	93 (86.9)
Yes	18 (11)	69 (66; 73)	29.38 (24; 34.63)	4 (7)	14 (13.1)
*P*-value		*P*=0.298^a^	*P*=0.920^a^	*P*=0.237^b^	

^a^Mann-Whitney U test; ^b^Pearson’s chi-squared test

BMI=body mass index; IQR=interquartile range

**Fig. 1:**
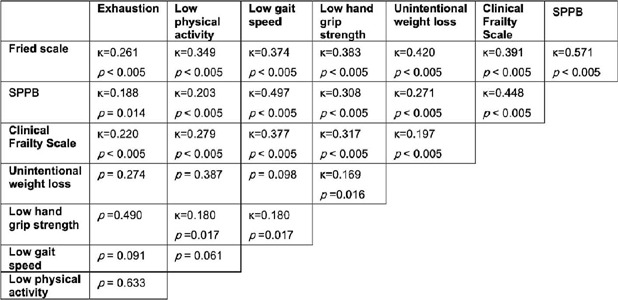
Fleiss’ kappa test results demonstrating lack of agreement between frailty assessment tools on identifying patients as frail. SPPB=Short Physical Performance Battery.

**Fig. 2:**
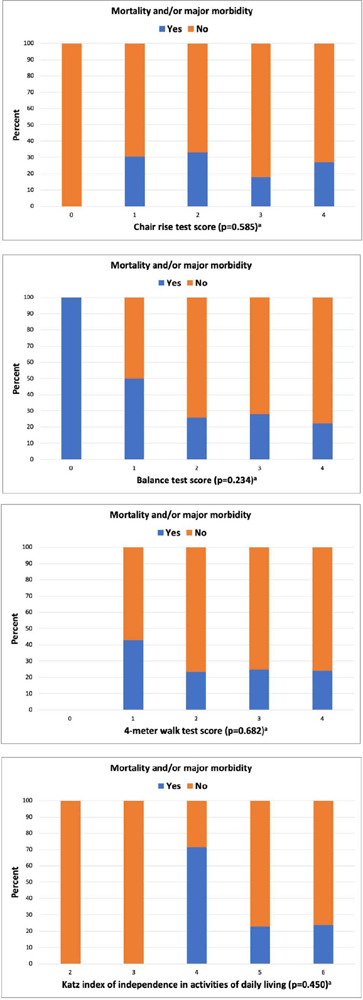
Association of (A) chair rise test, (8) balance test, (C)4-meter walk test, (0)Katz index with postoperative mortality and/or major morbidity a Mann-Whitney U test.

There was variability between frailty tools regarding frailty prevalence. Thirty-eight (23.2%) patients were categorized as frail by Fried’s scale, 42 (25.6%) by SPPB, and 70 (42.7%) by CFS (Table 4). Moreover, the agreement between Fried’s scale, SPPB, and CFS regarding frailty diagnosis was less than good (*P*<0.005) (Figure 1). Study population was relatively independent in daily living activities with a median Katz index of 6 (IQR, 5 to 6). Albumin data was missing for 30 participants. Remaining 134 patients had a median albumin level of 4.04 g/dl (IQR, 3.68 to 4.36) (Table 4). Among cardiac surgery risk scores and frailty assessment tools, only EuroSCORE II and albumin were significantly associated with the primary endpoint (*P*=0.045 and *P*=0.031, respectively) (Table 5, Figure 2). An albumin level of 3.84 g/dl had the highest sensitivity and specificity to predict a composite of mortality and/or major morbidity (area under curve = 0.637). Using the cutoff of albumin < 3.84 g/dl, we identified 43 (26.2%) patients as frail. Patients in the lower albumin group had a significantly increased relative risk of experiencing the primary endpoint with a risk ratio of 2.398 (95% confidence interval, 1.327-4.336) (Table 5). Surgical risk scores and frailty tests were not associated with any of the secondary endpoints (*P*>0.05).

**Table 4:** Frailty scores.

Fried scale, n (%)	
Non-frail	126 (76.8)
Frail	38 (23.2)
Short Physical Performance Battery, n (%)	
Not frail	122 (74.4)
Frail	42 (25.6)
Clinical Frailty Scale, n (%)	
Not frail	94 (57.3)
Frail	70 (42.7)
Unintentional weight loss, n (%)	
Not frail	132 (80.5)
Frail	32 (19.5)
Low hand grip strength, n (%)	
Not frail	102 (62.2)
Frail	62 (37.8)
Low gait speed, n (%)	
Not frail	83 (50.6)
Frail	81 (49.4)
Low physical activity, n (%)	
Not frail	83 (50.6)
Frail	81 (49.4)
Exhaustion, n (%)	
Not frail	134 (81.7)
Frail	30 (18.3)
Balance test, median (IQR)	4 (3 to 4)
4-meter walk test, median (IQR)	3 (3 to 4)
Chair rise test, median (IQR)	4 (3 to 4)
Katz index, median (IQR)	6 (5 to 6)
Albumin (g/dl), median (IQR)	4.04 (3.68 to 4.36)

IQR=interquartile range

**Table 5:** Relationship of cardiac surgical risk models and frailty assessment tools with the primary endpoint.

	Primary endpoint		*P*-value	Risk ratio	95% CI
	No	Yes			
EuroSCORE II, median (IQR)	2.09 (1.42; 3.77)	3.33 (1.89; 4.31)	0.045^a^		
STS-PROM, median (IQR)	1.35 (0.92; 2.30)	1.60 (0.94; 2.42)	0.150^a^		
STS-PROMM, median (IQR)	10.18 (7.52; 14.05)	11.15 (8.31; 18.99)	0.109^a^		
Fried scale, n (%)			0.831^b^	1.070	0.579-1.976
Non-frail (0 to 2)	95 (75.4)	31 (24.6)			
Frail (3 to 5)	28 (73.7)	10 (26.3)			
SPPB, n (%)			0.836^b^	1.065	0.587-1.932
Not frail (9 to 12)	92 (75.4)	30 (24.6)			
Frail (0 to 8)	31 (73.8)	11 (26.2)			
Clinical Frailty Scale, n (%)			0.101^b^	1.555	0.915-2.642
Not frail (0 to 4)	75 (79.8)	19 (20.2)			
Frail (5 to 9)	48 (68.6)	22 (31.4)			
Unintentional weight loss, n (%)			0.999^b^	1	0.512-1.952
Not frail	99 (75)	33 (25)			
Frail	24 (75)	8 (25)			
Hand grip strength, n (%)			0.577^b^	1.165	0.682-1.990
Not frail	78 (76.5)	24 (23.5)			
Frail	45 (72.6)	17 (27.4)			
Gait speed, n (%)			0.787^b^	1.076	0.633-1.829
Not frail	63 (75.9)	20 (24.1)			
Frail	60 (74.1)	21 (25.9)			
Low physical activity, n (%)			0.928^b^	0.976	0.574-1.659
Not frail	62 (74.7)	21 (25.3)			
Frail	61 (75.3)	20 (24.7)			
Exhaustion, n (%)			0.816^b^	1.083	0.558-2.102
Not frail	101(75.4)	33 (24.6)			
Frail	22 (73.3)	8 (26.7)			
Albumin, n (%)			0.003^b^	2.398	1.327-4.336
Not frail	76 (83.5)	15 (16.5)			
Frail	26 (60.5)	17 (39.5)			

^a^Mann-Whitney U test; ^b^Pearson’s chi-squared test

CI=confidence interval; EuroSCORE II=European System for Cardiac Operative Risk Evaluation II; IQR=interquartile range; SPPB=Short Physical Performance Battery; STS-PROM=Society of Thoracic Surgeons-predicted risk of mortality; STS-PROMM=Society of Thoracic Surgeons-predicted risk of mortality and morbidity

In order to investigate whether addition of a frailty parameter to cardiac surgical risk scoring systems would increase prognostic power, a diagnostic model was created by combination of EuroSCORE II and serum albumin, and the new model’s ability to predict the primary endpoint was assessed. Whereas EuroSCORE II’s R-squared value increased from 0.07 to 0.144 after addition of albumin, the new model was not associated with the primary outcome (*P*=0.571).

## DISCUSSION

The results of the present study indicate that assessment of frailty beforecardiacsurgeryinelderlypatientshasthepotentialtoimprove efficiency of risk prediction and decision-making processes in this delicate patient population. This is in concordance with previous research demonstrating that frailty assessment has incremental predictive value over widely utilized risk scoring systems including the STS and EuroSCORE^[14,15,16,17,18,19]^. In the FRAILTY-AVR study, which included 1020 patients across 14 centers and three countries, Afilalo et al.^[14]^ investigated the value of frailty to predict all-cause mortality and disability one year after surgical and transcatheter aortic valve replacement (TAVR). The authors concluded that frailty, measured by the Essential Frailty Toolset, was a strong predictor of mortality and disability^[14]^. Similarly, in a study of 400 adults aged ≥ 74 years, Sündermann et al.^[16]^ observed that Comprehensive Assessment of Frailty (CAF) toolset accurately identified patients at risk of 30-day mortality following cardiac surgical procedures. In the one-year follow-up of the same cohort, a simplified version of the CAF toolset also predicted adverse events at mid-term^[17]^.

Albumin was the only frailty tool that predicted early postoperative mortality and morbidity in elective cardiac surgery patients ≥ 65 years of age. This correlates with recent evidence reporting that lower preoperative albumin is associated with increased risk of adverse outcomes in adults undergoing cardiac procedures^[12,13,14]^. In a retrospective analysis of 470 patients, Hebeler et al.^[12]^ revealed that among a set of frailty assessment tools, albumin was the only frailty marker associated with one-year mortality in patients undergoing TAVR. Similarly, in a retrospective study by Forcillo et al.^[13]^ including 361 high- and extreme-risk TAVR patients, albumin was the only frailty test that was predictive of 30-day all-cause mortality.

We also demonstrated that addition of albumin as a frailty marker to EuroSCORE II risk model did not increase the model’s ability to predict early postoperative mortality and morbidity, however has the potential to do so; a finding that supports previous research reporting improved prognostic power after inclusion of a frailty parameter to traditional risk scores^[1,15,16]^. This finding is especially important since EuroSCORE II includes poor mobility as a risk variable, but not as a measure of frailty, rather as a measure of disability secondary to musculoskeletal or neurological dysfunction^[3]^. Whereas there is evidence that poor mobility has superior predictive value for adverse outcomes after TAVR compared to several frailty indices, it should be noted that poor mobility as defined by EuroSCORE II is not a frailty measure^[3,20]^. The STS has been collecting five-meter gait speed data as a potential frailty marker for over a decade, however, gait speed variable has not been included in the current version of the STS risk model due to missing data in 95% of patients^[1]^. Performance-based tests such as gait speed and hand grip strength are time-consuming, often require specialized environment and equipment, and may not always be suitable for the practicing clinician to perform^[21,22,23]^. Albumin, a cheap, quick, easy-to-perform biomarker of frailty, may be considered as an adjunct or alternative to poor mobility and five-meter gait speed test for inclusion in the EuroSCORE II and STS adult cardiac surgery risk models. Albumin may also be useful in the follow-up of prehabilitation of frail adults before cardiac surgery^[24]^. By all means, inclusion of a convenient frailty marker to current cardiac surgical risk scoring systems would be significantly beneficial for the elderly patients, especially those with severe aortic stenosis who need a decision between surgery and TAVR^[14]^. Even though frailty, disability, and comorbidity are distinct entities, they cannot be entirely separated from each other. Frailty is primarily a concern of older age, and disabilities and comorbidities increase in incidence with older age. A frail person is more prone to become disabled or comorbid, and vice versa. Therefore, it is reasonable to assume that frailty tools, which stem from a frailty phenotype and assess frailty as a distinct entity, are more useful in community-based research rather than clinical research^[22,25,26,27]^. This might be the explanation why phenotypic approaches such as Fried’s scale and SPPB failed to predict postoperative adverse outcomes in our population of patients with cardiac disease. In addition, frailty tests that measure performance are susceptible to patient manipulation. It was the authors’ observation that some patients tried to reflect themself better or worse than they actually are. Albumin is more resistant to such manipulation. With this regard, it should be noted that there are several tools to assess frailty based on various approaches. The most appropriate tool for a given setting and patient population should be utilized and included in the surgical evaluation process as an indicator to decide whether a patient is a suitable candidate for surgery or not.

A lack of significant association or correlation between demographics and the primary endpoint indicates that frailty was an age- and sex-independent predictor of adverse outcomes in our cohort. Besides, EuroSCORE II and STS-PROM underestimated mortality, and STS-PROMM underestimated composite of mortality and morbidity. This correlates with a recent study by Taleb-Bendiab et al.^[28]^, which reported that both EuroSCORE II and STS score significantly underestimated postoperative risk in 1229 elderly patients undergoing cardiac surgery. These findings, along with other reports, suggest that cardiac surgical risk stratification of elderly patients should include a measure of frailty, independently of chronological age^[1,29,30]^. This also explains why we observed higher-than-expected mortality and morbidity rates in our study population.

Body mass index was not associated with postoperative outcomes. This is in accordance with research reporting that frailty is not synonymous with low body weight^[6,27]^. An obese patient may be sarcopenic with reduced muscle mass. Therefore, low body mass index should not be used as a sign of frailty.

### Limitations

There are several limitations to the present study. Sample size was not large enough to assess association of frailty with mortality and morbidities as isolated endpoints. Therefore, a composite endpoint was chosen. Due to observational design, albumin data was missing in 30 participants. This did not cause a concern regarding statistical power, however, resulted in a reduction in sample size during creation of combined diagnostic models. Despite consecutive sampling, EuroSCORE II of the patient cohort was on the lower spectrum. This might have created a spectrum bias and explain why EuroSCORE II had lower than expected predictive value. A limited number of frailty tools were investigated due to observational design and limited funding. Follow-up duration was short, so we were not able to evaluate the predictive value of frailty on long-term outcomes after cardiac surgery. Finally, patients undergoing TAVR were not included in the analysis.

## CONCLUSION

Among frailty tests, serum albumin measurement had the highest predictive value for a composite of early postoperative mortality and morbidity in elderly patients undergoing elective cardiac surgery. Addition of albumin measurement as a frailty marker to EuroSCORE II cardiac surgical risk scoring system has the potential to improve EuroSCORE II’s ability to predict postoperative adverse outcomes. Further investigation with a larger sample from a wider spectrum of preoperative risk is warranted.

## Abbreviations, Acronyms & Symbols

BMI: Body mass index
CABG: Coronary artery bypass grafting
CAF: Comprehensive Assessment of Frailty
CFS: Clinical Frailty Scale
CI: Confidence interval
EuroSCORE II: European System for Cardiac Operative Risk Evaluation II
IQR: Interquartile range
NYHA: New York Heart Association
PROM: Predicted risk of mortality
PROMM: Predicted risk of mortality and major morbidity
SPPB: Short Physical Performance Battery
STS: Society of Thoracic Surgeons
TAVR: Transcatheter aortic valve replacement
